# Inversion of diffraction data for amorphous materials

**DOI:** 10.1038/srep33731

**Published:** 2016-09-22

**Authors:** Anup Pandey, Parthapratim Biswas, D. A. Drabold

**Affiliations:** 1 Department of Physics and Astronomy, Condensed Matter Surface Science Program, Ohio University, Athens OH 45701, USA; 2 Department of Physics and Astronomy, The University of Southern Mississippi, Hattiesburg MS 39406, USA; 3 Department of Physics and Astronomy, Ohio University, Athens OH 45701, USA

## Abstract

The general and practical inversion of diffraction data–producing a computer model correctly representing the material explored–is an important unsolved problem for disordered materials. Such modeling should proceed by using our full knowledge base, both from experiment and theory. In this paper, we describe a robust method to jointly exploit the power of *ab initio* atomistic simulation along with the information carried by diffraction data. The method is applied to two very different systems: amorphous silicon and two compositions of a solid electrolyte memory material silver-doped GeSe_3_. The technique is easy to implement, is faster and yields results much improved over conventional simulation methods for the materials explored. By direct calculation, we show that the method works for both poor and excellent glass forming materials. It offers a means to add *a priori* information in first-principles modeling of materials, and represents a significant step toward the computational design of non-crystalline materials using accurate interatomic interactions and experimental information.

On the eve of the First World War, William Lawrence Bragg and his father, William Henry Bragg, exposed crystalline solids to X-rays and discovered what we now call “Bragg diffraction”, strong reflection at particular incident angles and wavelengths. These “Bragg peaks” were sharply defined and, when analyzed with a wave theory of the X-rays, led to clear evidence of order in the crystalline state^1^. By analyzing the diffraction angles at which the peaks appeared and the wavelength of the X-rays, the full structure of the crystal could be ascertained. In the language of modern solid state physics, the X-ray structure factor of a single crystal consists of a sequence of sharp spikes, which are broadened in a minor way by thermal effects. The information obtained from this palisade of delta functions, arising from a crystal, is sufficient for the determination of lattice structure. The rapid development of X-ray Crystallography in the past several decades had made it possible to successfully determine the structure of complex protein molecules, with more than 10^5^ atoms, leading to the formation of a new branch of protein crystallography in structural biology^2^.

In contrast with crystals, amorphous materials and liquids have structure factors that are smooth, and thus contain far less specific information about structure. The lack of sharp peaks principally originates from the presence of local atomic ordering in varying length scales, and no long-range order in the amorphous state. The resulting structure factor is one-dimensional and is effectively a sum rule that must be satisfied by the three-dimensional amorphous solids. This presents a far more difficult problem of structural determination of amorphous solids that requires the development of new tools and reasoning. A natural approach to address the problem is to carry out computer simulations, either employing molecular dynamics or Monte Carlo, with suitable interatomic potentials. We have called this approach the “simulation paradigm”^3^ elsewhere. By contrast, the other limit is to attempt to invert the diffraction data by “Reverse Monte Carlo” (RMC) or otherwise without using any interatomic potential but information only^4^,^5^. This we have called the “information paradigm”^3^. The information paradigm in its purest form produces models reproducing the data using a random process. These models tend to be maximally disordered and chemically unrealistic. The information paradigm is closely related to the challenge of Materials by Design^6^,^7^, for which one imposes external constraints to incorporate additional information on a model to enable a set of preferred physical properties that are of technological utility.

Neither paradigm is ideal, or even adequate. The simulation paradigm is plagued by severe size and time-scale limitations that misrepresent the real process of forming a glass, not to mention imperfect interatomic interactions. For amorphous materials with no or weak *glass-forming* ability, either approach is rather desperate, and leads to the formation of unrealistic models with too many structural defects in the networks. In this paper we introduce *ab initio Force Enhanced Atomic Refinement* (AIFEAR). A preliminary trial of the algorithm using only empirical potentials recently appeared^8^.

Others have undertaken related approaches^9^,^10^,^11^,^12^,^13^,^14^,^15^,^16^,^17^. By including ‘uniformity’ as a constraint for the refinement of models, Goodwin and coworkers showed their Invariant Environment Refinement Technique^11^ to produce improved models of a-Si and other systems. A liquid-quench procedure, combined with a hybrid Reverse Monte Carlo approach, which incorporates both experimental and energy-based constraints has been employed by Opletal and coworkers^12^. A similar approach via hybrid RMC with empirical bonded and non-bonded forces was used by Gereben and Pusztai to study liquid dimethyl trisulfide^18^. Likewise, by refining the initial interatomic empirical potential-energy function and fitting the input experimental structure-factor data, empirical potential structure refinement has been quite successful in predicting liquid structures^14^. An alternative approach, experimentally constrained molecular relaxation, which incorporates experimental information in first-principles modeling of materials in a ‘self-consistent’ manner^16^ was discussed in refs 15 and 16. Recently, a means for including *electronic a priori* information has also appeared^17^. These methods have all contributed significantly to the field, yet they have limitations such as employing empirical potentials of limited reliability^8^,^12^, or unacceptable convergence properties^15^,^16^. *A general and successful framework for inverting solid state diffraction data does not exist. AIFEAR is a major step toward this important goal*.

We begin with some definitions. If *V*(*X*_1_ … *X*_*n*_) is the energy functional for atomic coordinates {*X*_*i*_} and *χ*^2^ measures the discrepancy between diffraction experiment and theory, we seek to find a set of atomic coordinates {*X*_*i*_} with the property that *V* = minimum and *χ*^2^ is within experimental error. AIFEAR is a simple iterative process consisting of (i) producing a structural model at random (at a sensible but not necessarily exact density, which may not be available), (ii) invoking *N* accepted moves within conventional RMC^19^ followed by *M* conjugate-gradient (CG) steps using *ab initio* interactions. We then iterate (ii) until convergence. The final results do not depend heavily on the numerical values of *N* and *M*, which were chosen to be 1000 and 10, respectively, for the present work. For the examples discussed here, we find that significantly fewer *ab initio* force calls are needed for AIFEAR than for an *ab initio* ‘melt-quench’ simulation. In addition, AIFEAR avoids the problem of relative weighting of *V* and *χ*^2^ in a penalty or target energy functional as in hybrid approaches developed elsewhere^12^,^20^. If the density of the material is unknown, it is straightforward to carry out the simulation at zero pressure (with variable cell geometries) in the CG loop, and simply pass the modified supercell vectors back to the RMC loop.

To illustrate the efficacy of this new approach, we begin with a persistently vexing problem: the structure of amorphous Si which is particularly difficult because the network is over-constrained^21^,^22^ and it is not a glass former. The only methods that yield really satisfactory results are the Wooten-Weaire-Winer (WWW)^23^ and Activation Relaxation Technique^24^ methods. Structural and electronic experiments reveal that coordination defects in good quality material have a concentration less than a part in 1000. As such, a satisfactory model should have at most a few percent (or less) defects. Inversion methods like RMC and *ab initio* melt-quench both produce unsatisfactory models with far too many coordination defects compared to experiments. In this illustration, we employ the local-orbital-based density functional code SIESTA for the calculation of *ab initio* forces, but the approach is easily implemented with *ab initio* total-energy plane-wave codes as we show in the next example.

We began by preparing three 216-atom models of *a*-Si, at the experimental density^25^ of 2.33 g.cm^−3 ^, using (1) RMC, (2) melt-quench, and (3) AIFEAR. The starting atomic configurations were chosen to be *random*, and the diffraction data from ref. 23 were employed in RMC and AIFEAR. The structural properties of *a*-Si, obtained from these models, are summarized in Fig. 1. For a discussion on convergence and comparisons to other calculations, see the Methods section. RMC produces a highly unrealistic model, far from the accepted tetrahedral network topology, as seen in Fig. 1. Melt-quench, while better, still produces far too many coordination defects. By contrast, AIFEAR produces a nearly perfect tetrahedral structure, with 99.07% fourfold coordination, and a bond-angle distribution close to that of a WWW model. In comparing the bond-angle distributions (from AIFEAR with that of from WWW), one must take into account the fact that *ab initio* interactions tend to produce a slightly wider bond-angle distribution than the highly artificial WWW (Keating spring) interactions.

We wish to emphasize that the starting configuration used in AIFEAR was *random*, so that one can logically infer that a combination of atomic-radial-correlation data and DFT interactions leads to an almost perfect tetrahedral network as illustrated in Fig. 1. Table 1 lists the key structural properties of the model, along with the total energy per atom. In the Methods section, we report the detailed convergence of total energy *E* and *χ*^2^. In the Supplementary Materials, we also offer an animation of the convergence of AIFEAR by showing the formation of a tetrahedral network as the simulation proceeds with the disappearance of coordination defects.

For a challenging and timely example, we have also studied the solid electrolyte material Ag_*x*_(GeSe_3_)_1−*x*_. This is a chemically complex system with important applications to conducting bridge computer FLASH memory devices, which are of considerable fundamental and technological interest. We employ the same scheme as for *a*-Si, but with *ab initio* interactions from the plane-wave DFT code VASP^26^,^27^,^28^, with 135 and 108 atoms in a unit cell of length 15.923 Å and 15.230 Å for *x* = 0.05 and *x* = 0.077, respectively. These values correspond to the densities of 4.38 g.cm^−3^ and 4.04 g.cm^−3^ for the models with 5% and 7.7% Ag, respectively. For *x* = 0.05, both the structure-factor data and density of 4.38 g.cm^−3^ are taken from the work of Piarristeguy and Pradel^29^. For *x* = 0.07, we have used the RDF data provided by Zeidler and Salmon^30^, and a density of 4.04 g.cm^−3^ was obtained from a zero-pressure conjugate-gradient relaxation using VASP. For completeness, we have also studied a melt-quench model of *x* = 0.077 as described in the Methods section. The melt-quench model (in Fig. 2a) shows significant discrepancies with experiments: the first sharp diffraction peak (FSDP) near 1 Å^−1^ is absent, and there are significant inconsistencies in the structure factor at high *k* values. The FSDP is an indicator of medium range order, a signature of structural correlations between the tetrahedral GeSe structural building blocks of the glass. By contrast, the AIFEAR model captures all the basic characteristics of the structure factor, including the FSDP (in fact, it slightly *overfits* the FSDP). We show that the method has similar utility in either real or *k* space, using *S*(*k*) for the first composition and *g*(*r*) for the second. Figure 2 shows the structure factors and radial distribution functions obtained from AIFEAR and melt-quench simulations, and compares with the experimental data from neutron diffraction measurements^29^,^30^.

The GeSeAg systems are of basic interest as solid electrolytes. One of the most interesting questions pertains to the dynamics of Ag atoms, which are sufficiently rapid that they can be tracked even in first-principles molecular-dynamics simulations^31^. The fast Ag dynamics have led to the invention of conducting bridge Random Access Memory^32^,^33^. As this dynamics appears to be of trap-release form^31^, the structure, including features like medium range order, and associated energetics may be expected to play a key role in the silver hopping. The 7.7% Ag composition is near to a remarkable and abrupt ionic mobility transition^34^,^35^. Dynamical simulations are currently underway to determine the role of the structure in this dynamics.

The following features of Ag_*x*_(GeSe_3_)_1−*x*_ glasses have been observed in the AIFEAR model: 1) the Ge-Se correlation is not affected by an increase in Ag content: Ge(Se_1/2_)_4_ tetrahedra remain the fundamental structural units in the network. 2) Ge-Ge correlations, greatly affected by Ag doping, are revealed by the shift in Ge-Ge nearest-neighbor distance from 3.81 Å in Ag = 0%^29^ to 2.64 Å and 2.56 Å in Ag = 5% and Ag = 7.7%, respectively 3) the Ag-Se correlation peak is near 2.66 Å for both the systems, which is consistent with the experimental work of Zeidler^30^ and others^29^. 4) The Se-Se coordination number for 5% and 7.7% Ag are 1.12 and 0.83 (0.81 from experimental data^30^), respectively. This is consistent with the observed phenomena of decrease in Se coordination with the increase in Ag concentration^29^.

Beside retaining the important chemical features of the network, the AIFEAR model is superior to the melt-quench model by the manifestation of a prominent FSDP (cf. Fig. 2a), a signature of medium range order in these materials. Absence of the FSDP indicates the lack of structural correlations in the Ge(Se_1/2_)_4_ tetrahedra, which is less prominent for low Ag concentration. Also, the energy of the AIFEAR model for x = 0.077 is 0.02 eV/atom less than the melt-quench model (see Fig. 3b).

It is important and promising that in the GeSeAg systems, as in *a*-Si, AIFEAR is not a greedy optimization scheme, as it is evidently able to unstick itself (for example in Fig. 3b) near 400 steps, there is a dramatic and temporary increase in *χ*^2^, which then enabled the system to find a new topology which produced further reduction of both *χ*^2^ and energy. A similar, if less dramatic, event is indicated in Fig. 3a around step 1100. The Monte-Carlo moves robustly explore the configuration space and are not so prone to getting trapped as in MD simulations, and yet the chemistry is properly included in the *ab initio* relaxation loop.

In conclusion, we have introduced a new and practical method that enables the joint exploitation of experimental information and the information inherent to *ab initio* total-energy calculations, and a powerful new approach, to the century-old problem of structural inversion of diffraction data. The method is simple and robust, and independent of the systems, the convergence of which has been readily obtained in two highly distinct systems, both known to be challenging and technologically useful. By direct calculation, we show the network topology of a-Si, implied by the atomic pair correlations and accurate total energies, is: structurally similar to WWW models, including the bond-angle distribution. Using only the total structure-factor (or pair-correlation) data and SIESTA/VASP, we obtain models of unprecedented accuracy for a difficult test case (*a*-Si) and a technologically important memory material (GeSe_3_Ag). The inclusion of *a priori* experimental information emphasized here may also be developed into a scheme to include other information for materials optimization. It is easily utilized with any interatomic potentials, including promising current developments in “machine learning”^36^. The method is unbiased in the sense that it starts from a completely random configuration and explore the configuration space of a total-energy functional aided by additional experimental information to arrive at a stable amorphous state. Beside these attributes, it requires fewer force calls to the expensive *ab initio* interactions.

## Methods

As described in the main text, AIFEAR jointly minimizes the configurational energy *V* and the cost function (see refs 4 and 37)


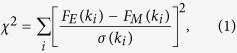


where *F*_*E*/*M*_(*k*_*i*_) is the experimental/model structure factor, and *σ* (*k*_*i*_) is the error associated with the experimental data for wave vector *k*_*i*_. To undertake this program, (i) we begin with a random model, (ii) invoke *M* RMC accepted moves followed by *N* conjugate-gradient steps to optimize the total energy. We have found *M* = 1000 and *N* = 10 to be satisfactory for the materials of this paper. The process (ii) is repeated until the desired accuracy of *δχ*^2^ ≈ 0.1 and a force tolerance of *δf* ≈ 0.02 eV/Å is attained. All that is required are RMC and total-energy codes and an appropriate driver program connecting them.

### Amorphous Si

**Figure 4:**
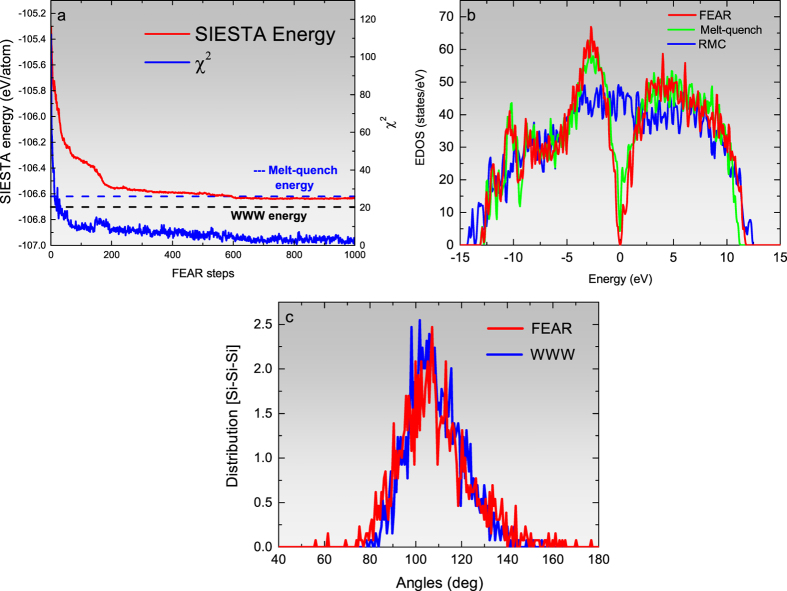
Results for 216-atom *a*-Si: (**a**) The variation of cost function and total energy with the number of AIFEAR steps. (**b**) Electronic density of states (EDOS) for RMC, melt-quench and AIFEAR models with the Fermi level at 0 eV. (**c**) The bond-angle distribution from AIFEAR compared to that of WWW (see Table 1 for details).

Initially, conventional RMC (i.e. without any constraint) runs were performed using the RMCProfile software^19^ for a *random* starting configuration of 216-atom *a*-Si with a cubic box of side 16.281 Å corresponding to the density 2.33 g.cm^−3^. The maximum step length of the RMC moves for Si atoms is chosen to be 0.05 Å. In a parallel simulation, the same starting configuration is taken through a process of melt-quench using the density-functional code SIESTA^38^ with single-*ζ* basis under Harris functional scheme^38^ within the local density approximation. The total-energy and force calculations are restricted to the Γ point of the supercell Brillouin zone. After melting at 2300 K, the liquid structure was quenched to 300 K at a rate of 240 K/ps. Each step was followed by the equilibration of the system for 2000 time steps. To ensure the reproducibility of the FEAR method, we have generated 10 *a*-Si models starting from random configurations and the models yielded 4-fold coordination always exceeding 96%. Details of convergence and comparison to the best available WWW model is provided in Fig. 4. The elimination of defects is chronicled in an animation provided in the Supplementary Materials.

### Chalcogenide glasses

The experimental structure factors data taken from the work of Piarristeguy *et al*.^29^ for 5% Ag and the pair distribution function (PDF) was obtained from the work of Zeidler and Salmon^30^ for 7.7% Ag. For ab initio interactions, we used the plane-wave DFT code VASP^26^,^27^,^28^ using projected augmented plane waves (PAW)^39^ with Perdew-Burke-Ernzerhof (PBE) exchange-correlation functional^40^ and a plane-wave cutoff of 312.3 eV. All calculations were carried out at Γ point. The *random* starting configurations of 5% and 7.7% Ag-doped GeSe_3_Ag were subjected to *ab initio* FEAR. The 5% Ag-doped GeSe_3_Ag *ab initio* FEAR model is compared to the melt-quench model of the identical system of Piarristeguy and co-workers^29^. The melt-quench model of 7.7% Ag-doped GeSe_3_ is prepared by melting the same starting configuration at 1400 K for 10,000 steps, followed by a quenching to 300 K at the rate of 100 K/ps, and then by equilibrating at 300 K for another 5000 steps. To estimate the density of the equilibrated system, the volume of the simulation cell was relaxed. A final relaxation at zero pressure was employed, which yielded a density of 4.04 g.cm^−3^. A time step of 1.5 fs was used for the production of melt-quenched models in MD simulations.

**Figure 5:**
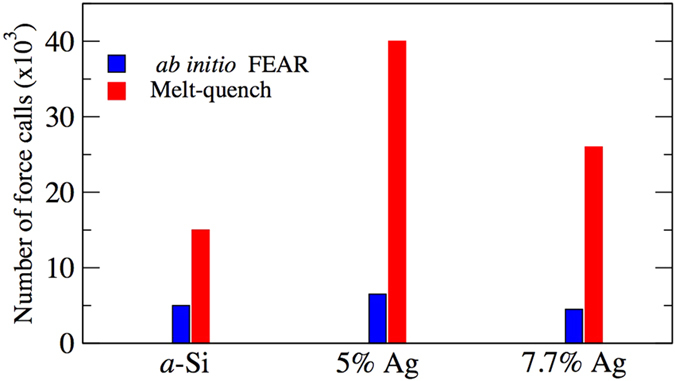
Comparison of number of force calls in *ab initio* FEAR with melt-quench simulations for *a*-Si, and 5% and 7.7% Ag-doped GeSe_3_. Note that the number of force calls in melt-quench simulations vary considerably for different systems.

We have included a comparison of the number of force calls in the various simulations in Fig. 5. It is evident from Fig. 5 that AIFEAR offers a significant computational advantage, with fewer force calls to the expensive *ab initio* codes.

## Additional Information

**How to cite this article**: Pandey, A. *et al*. Inversion of diffraction data for amorphous materials. *Sci. Rep.* **6**, 33731; doi: 10.1038/srep33731 (2016).

## Supplementary Material

Supplementary Information

Supplementary Video S1

Supplementary Video S2

Supplementary Video S3

## Figures and Tables

**Figure 1:**
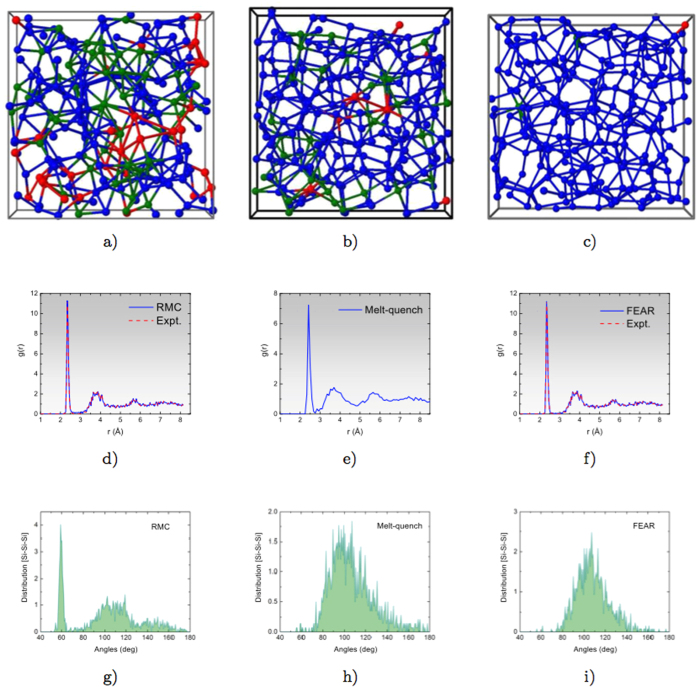
Top: A 216-atom model of *a*-Si obtained from (**a**) RMC, (**b**) melt-quench and (**c**) AIFEAR simulations. Silicon atoms with a coordination number of 3, 4 and 5 are shown in green, blue and red colors, respectively. Center: The radial distribution function (RDF) for the (**d**) RMC, (**e**) melt-quench and (**f**) AIFEAR models. Bottom: The bond-angle distributions for the models as indicated in the figure. See supplementary materials for animations showing the formation of three-dimensional network structure and the corresponding evolution of the radial and coordination-number distributions.

**Figure 2:**
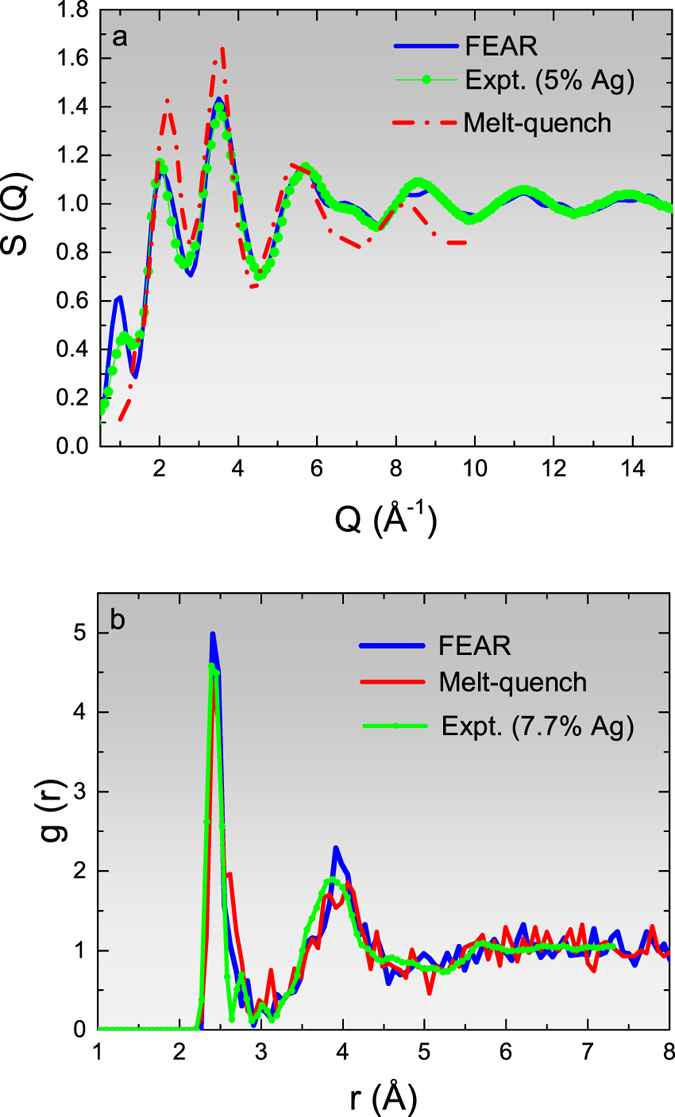
(**a**) Structure factors of (GeSe_3_)_1−*x*_Ag_*x*_ [*x* = 0.05] from AIFEAR. Experimental data, from neutron diffraction measurements, are shown for comparison^29^. Melt-quench data are from Pradel *et al*.^29^ (**b**) The radial distribution function of (GeSe_3_)_1−*x*_Ag_*x*_ [*x* = 0.077] from AIFEAR and melt-quench simulations. Experimental RDF shown here are from Zeidler *et al*.^30^.

**Figure 3:**
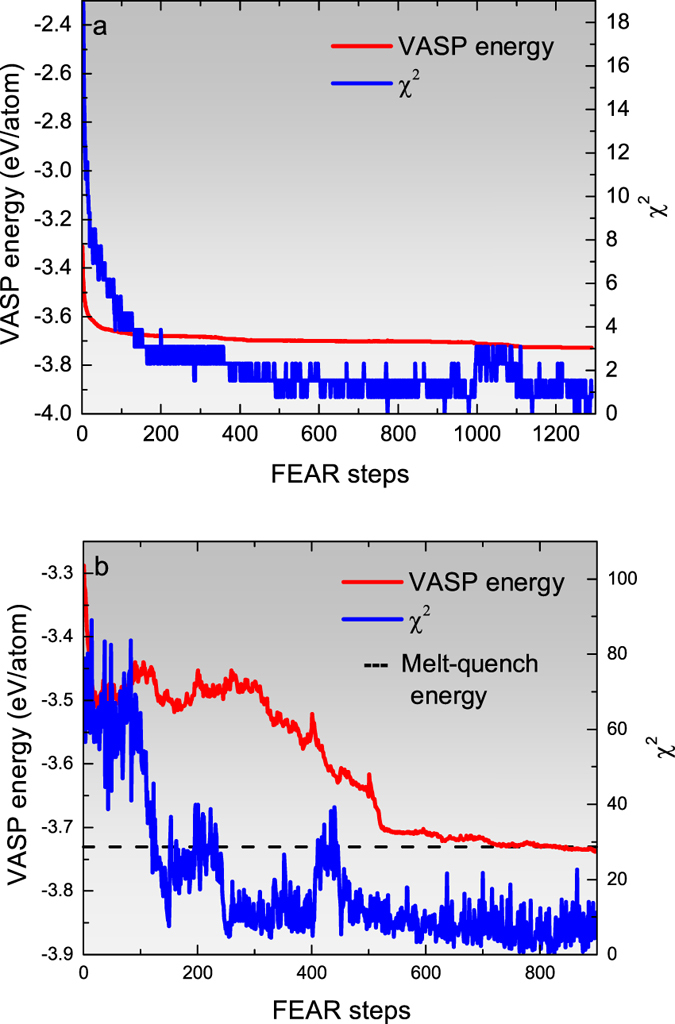
Total energy per atom and the cost function (*χ*^2^) versus AIFEAR steps for two models with (**a**) 5% and (**b**) 7.7% Ag-doped GeSe_3_. The melt-quench energy for the 7.7% Ag model is indicated for comparison.

**Table 1:** Total energy and key structural properties of *a*-Si models.

	RMC	Melt-quench	AIFEAR	WWW
4-fold Si (%)	27	80	99.07	100
SIESTA energy (eV/atom)	3.84	0.08	0.03	0.00
Average bond angle (RMS deviation)	101.57° (31.12°)	107.04° (20.16°)	108.80° (14.55°)	108.97° (11.93°)

The energy per atom is expressed with reference to the energy of the WWW model.
