# Supplementary material for: Inversion of diffraction data for amorphous materials
Source: Sci Rep. 2016 Sep 22;6:33731. doi: 10.1038/srep33731 (PMC5031976; doi:10.1038/srep33731)
Supplement: Supplementary Information [file srep33731-s1.pdf]

Supplementary Information for "Inversion of diffraction data for amorphous materials", by A. Pandey, P. Biswas and D. A. Drabold

There are three video files that we have uploaded as Supplementary Materials supporting this manuscript. The file names and descriptions follow.

movie_fear_strc_evol.mov: *Evolution of FEAR and the elimination of defects in a-Si. Colour-coding is explained in the video.*

rdf_evol.mov: *This animation depicts the evolution of the radial distribution function for a-Si with ab initio FEAR.*

RMC_CN.mov: *This animation depicts the evolution of the coordination number for a-Si using ab initio FEAR. A coordination number near four is expected for this system.*
